# Supplementary material for: Modelling the Cost-Effectiveness and Budget Impact of a Newborn Screening Program for Spinal Muscular Atrophy and Severe Combined Immunodeficiency
Source: Int J Neonatal Screen. 2022 Jul 20;8(3):45. doi: 10.3390/ijns8030045 (PMC9326684; doi:10.3390/ijns8030045)
Supplement: Supplementary file 1 [file IJNS-08-00045-s001.zip › IJNS-1684816-Supplementary.pdf]

Table S1: Model parameter and expected values with ranges for Markov model

Parameters	Expected values	Distribution	Low	High	Source
**Cost parameters**	US$ (A$)				
Screening test cost	$4.82 ($7)	Gamma	-10%	+10%	NBS Pilot
Repeat screening test cost	$6.89 ($10)	Gamma	7	25	NBS Pilot
SMA1 annual cost	$115,858 ($231,717)	Gamma	$50,000	$490,000	[34]
SMA2 annual cost	$76,234 ($152,469)	Gamma	$40,000	$360,000	[34]
SMA3 annual cost	$47,983 ($95,966)	Gamma	$20,000	$180,000	[34]
Gene therapeutic cost	$1,540,000 ($2,234,540)		$1,270,000	$2,100,000	[36]
Gene Tx follow-up cost	$158 ($230)				[37]
Gene Tx initial year cost	$4,312 ($4574)				[37,38]
Nusinersen Injection episode cost	$3,731 ($5,414)				[37,38]
Nusinersen loading cost	$318,164 ($461,656)				[2,39]
Nusinersen maintenance cost	$119,311 ($173,123)		$79,939	$131,242	[2,39]
Nusinersen one dose cost	$75,810 (110,000)		$68,228	$83,390	[39]
Confirmatory diagnostic SCID testing cost (after positive screening test)	$2119 ($3,074)				[13]
Diagnostic SCID testing cost (without NBS)	$3446 ($5,000)				[13]
Pre-symptomatic SCID cost	$0 ($0)				Assumption
HSCT cost (early diagnosis)	$119,282 ($173,078)	Gamma	-10%	+10%	[13]
HSCT cost (late diagnosis)	$271,697 ($394,233)	Gamma	-10%	+10%	[13]
Treatment cost for SCID patient dying prior to HSCT	$178,923 ($259,617)				[13]
SCID Well treatment cost (per year)	$34 ($50)				[13]
SCID Moderate treatment cost (per year)	$24,052 ($34,900)				[13]
SCID Poor treatment cost (per year)	$12,873 ($18,679)				[13]
End-of-life costs (SCID Moderate & SCID Poor)	$41,841 ($60,712)				[13]

SCID Well productivity cost	$0 ($0)				Assumption
SCID Moderate productivity cost	$1,394 ($2,023)				[13]
SCID Poor productivity cost	$0 ($0)				Assumption
Netherlands, 2016 PPP Euros/US$	0.796				[24]
Australia, 2016 PPP AU$/US$	1.45				[24]
CPI inflation rate 2016 to 2019 AU$	0.0557				[40]
Discount rate	0.03		-	0.05	
**Outcome parameters**					
SMA INCIDENCE	0.000091	Beta	0.000065	0.000119	[41]
SMA 1 mortality	0.29	Beta	0.227	0.353	[42]
SMA 2 mortality	0.004	Beta	0.0025	0.0059	[43]
SMA 3 mortality	Population background mortality				
SMA phenotype (SMA1, SMA2, SMA3)	0.58, 0.29, 0.13				[43]
False negative % in screen	0.00000576				[25]
Screen retest due to non-amplification	0.012				[25]
Further test % in screen	0.0000854	Beta	0.000038	0.000134	[25]
SMA confirmation in re-test	1				[25]
**Motor milestone half-yearly transition**					
Non-sitter to sit in early gene therapy	0.43	Beta	0.35	0.50	[2,44]
Non-sitter to sit in late nusinersen Tx	0.45	Beta	0.40	0.50	[45,46]
Sit to stand in early gene therapy	0.84	Beta	0.80	0.87	[2,44]
Sit to stand in late nusinersen Tx	0.55				[45,46]
Stand to assisted walk in early gene therapy	0.74	Beta	0.70	0.78	[2,44]
Stand to assisted walk in late nusinersen Tx	0.21				[45,46]
Walk assisted to independent walk in early gene therapy	0.81	Beta	0.73	0.88	[2,44]
Walk assisted to independent walk in late nusinersen Tx	0.58				[45,46]
SCID incidence	0.00002	Beta	0.000012	0.000025	[3,47]
False Negative % in Screen (1-sensitivity)	0.005	Beta	0	0.01	[10]
False Positive % in Screen (1-specificity)	0.0003	Beta	0.0002	0.0008	[10]
% patients early diagnosed without NBS	0.2	Beta	0.1	0.3	[9]

Probability to survive until treatment (early diagnosis)	0.9423				[3]
Probability to survive until treatment (late diagnosis)	0.78				[10]
5-year survival (early diagnosis)	0.94	Beta	0.91	0.98	[48]
5-year survival (late diagnosis)	0.82	Beta	0.7	0.9	[48]
SCID Well after HSCT (early diagnosis; surviving subjects)	0.8				[13]
SCID Moderate after HSCT (early diagnosis; surviving subjects)	0.15				[13]
SCID Poor after HSCT (early diagnosis; surviving subjects)	0.05				[13]
SCID Well after HSCT (late diagnosis; surviving subjects)	0.5				[13]
SCID Moderate after HSCT (late diagnosis; surviving subjects)	0.3				[13]
SCID Poor after HSCT (late diagnosis; surviving subjects)	0.2				[13]
**Quality of life utility value**					
Non-sitter	0.02				[15,34,49]
Sit without support	0.11				[19,34,49]
Stand with assistance	0.25				[34]
Walk with assistance	0.38				[49]
Stand walk unaided	0.64				[49]
Utility value for presymptomatic SCID	0.95				Assumption
Utility value for SCID Well	0.95				[13]
Utility value for SCID Moderate	0.75				[13]
Utility value for SCID Poor	0.5				[13]
Utility value for HSCT	0.5				Assumption
Utility value for Deceased	0				Assumption
